# Differential Responses of Liver and Hypothalamus to the Nutritional Condition During Lactation and Adult Life

**DOI:** 10.3389/fphys.2020.00553

**Published:** 2020-06-05

**Authors:** Isabela Ramos Mariano, Laís Akemi Yamada, Renan Soares Rabassi, Vanessa Lara Rissi Sabino, Camila Bataglini, Silvia Carla Santana Ferreira Azevedo, Rosângela Fernandes Garcia, Maria Montserrat Diaz Pedrosa

**Affiliations:** Laboratory of Physiological Sciences and Hepatic Metabolism, Department of Physiological Sciences, State University of Maringá, Maringá, Brazil

**Keywords:** caloric restriction, refeeding, liver metabolism, litter size, hypothalamus

## Abstract

It was previously reported that liver glucose metabolism in rats under caloric restriction differs from that of freely-fed rats. This study hypothesized that these changes (1) were related to the expression of hypothalamic neuropeptides involved in metabolic control, and (2) were not a residual effect of litter size. To those purposes, liver glucose metabolism and hypothalamic expression of the orexigenic neuropeptides NPY (neuropeptide Y) and AgRP (agouti gene-related peptide); and of the anorexigenic neuropeptides POMC (pro-opiomelanocortin) and CART (cocaine- and amphetamine-related transcripts) were investigated. Male Wistar rats from two different litter sizes (G6 and G12, with 6 or 12 pups, respectively) were subjected to free feeding (GL, *ad libitum*), 50% caloric restriction (GR) or caloric restriction+*ad libitum* refeeding (GRL) until the age of 90 days. Biometric values were lower in GR than in GL, while in GRL they were totally or partially recovered. Blood glucose variation during the pyruvate tolerance test (PTT) was small in GR. During *in situ* liver perfusion, total, basal, and adrenaline-stimulated liver glucose outputs were high in GR, but additional glucose output in the presence of alanine was negligible. Refeeding (GRL) yielded values close to those of GL. Litter size did not consistently influence any of these variables. The expression of transcripts of the hypothalamic neuropeptides was responsive to feeding regimen, litter size and/or their interaction and differed from G6 to G12, while the metabolic changes of the liver were qualitatively equal in both GR. Therefore, the changes in glucose metabolism in the liver of rats under caloric restriction were not determined by either litter size or hypothalamic neuropeptide expression and were linked only to the prevailing feeding regimen of the adult animal.

## Introduction

Energy homeostasis is the biological process that coordinates food ingestion, energy expenditure, and related processes, resulting in maintenance of proper levels of energetically rich substrates in the organism. This physiological process is strongly dependent of several organs and brain structures, among which the liver and the hypothalamus, respectively, stand out.

The liver is the central organ of energy homeostasis and blood glucose regulation. It responds independently to blood glucose concentration (Moore et al., 1998; Wahren and Ekberg, 2007) and is the target of humoral and neural agents that control energy metabolism (Hoffman, 2007; Rui, 2014). Although blood glucose tends to increase during carbohydrate absorption by the gastrointestinal tract and to decrease during fasting due to continuous uptake, especially by the nervous system, the outcome of liver functional responses is a physiologically acceptable fluctuation of blood glucose.

Many signals related to the acute and chronic energy status of the organism, such as insulin, leptin, neural afferents from the gastrointestinal tract, liver and adipose tissue, are channeled to hypothalamic nuclei to integrate the actions of the central command centers with the peripheral effector organs (Picó et al., 2012; Spencer, 2013; Bouret, 2017; Ruud et al., 2017). Several hypothalamic areas have a relevant role in energy homeostasis: arcuate nucleus (ARC), paraventricular nucleus (PVN), dorsomedial hypothalamus (DMH), ventromedial hypothalamus (VMH), and lateral hypothalamic areas (LHA) (Ruud et al., 2017). The ARC contains orexigenic neurons that co-express Neuropeptide Y (NPY) and Agouti gene-Related Peptide (AgRP), in addition to anorexigenic neurons co-expressing Pro-Opiomelanocortin (POMC) and Cocaine- and Amphetamine-Related Transcripts (CART) (Picó et al., 2012; Bouret, 2017). It has been reported, for instance, that the expression and release of hypothalamic NPY are directly related to a deficit of energy stores, such as decreased fat, reduced blood glucose and/or signaling by metabolic compounds in the brain (Patterson and Abizaid, 2013).

Early nutrition is said to influence energy homeostasis, being described as a programming agent of the central control of energy metabolism. In rodents, most of the connectivity among hypothalamic neurons takes place after birth, so that nutritional interventions during lactation may cause several permanent hypothalamic alterations with long-lasting consequences. This has been named metabolic programming, which can be related to impaired energy homeostasis and systemic metabolism and can lead to inappropriate feeding behavior, obesity, type 2 diabetes mellitus and associated comorbidities in adult life (Bouret, 2012; Picó et al., 2012; Spencer, 2013; Ruud et al., 2017).

In rodent experimental models, one way of manipulating the perinatal nutritional condition is by changing the number of pups of the litter during lactation, which causes overnutrition (small litters) or undernutrition (large litters). In large litters, milk availability is diminished and body growth during lactation is hampered (Bouret, 2012; Picó et al., 2012).

Our research group has been reporting changes of liver glucose metabolism caused by caloric restriction since lactation (Malta et al., 2010; Vitoriano et al., 2011; Babata et al., 2014; Garcia et al., 2017). In those studies, rats from 6-pups litters fed *ad libitum* after lactation had decreased glucose output and enhanced gluconeogenesis during *in situ* liver perfusion after overnight fasting, as expected, because fasting decreases liver glycogen stores and favors gluconeogenesis (Rui, 2014). In contrast, rats under caloric restriction since birth (raised in 12-pups litters and food-restricted to 50% after lactation) had a completely different profile: high glucose output and almost undetected gluconeogenesis after overnight fasting (Malta et al., 2010; Vitoriano et al., 2011), very similar to animals in post-prandial state (Babata et al., 2014). In another investigation, free feeding after caloric restriction (refeeding) decreased adrenaline-stimulated glycogenolysis after overnight fasting (Garcia et al., 2017), suggesting that the changes in glucose metabolism were not due to litter size (that is, lactational nutrition), but to the chronic feeding regimen and, as such, reversible.

Regarding central effects, caloric restriction was reported to promote an increase of NPY in the ARC, which in turn increases parasympathetic activity and decreases sympathetic tone, a response similar to low levels of corticotropin-releasing hormone, CRH (Heinrichs et al., 1993). The parasympathetic innervation of the liver activates glycogen synthase and stimulates glycogen synthesis (Rui, 2014).

Based on these observations, it seemed possible that hypothalamic and autonomic alterations would explain the metabolic changes of the liver induced by caloric restriction and refeeding and reveal a link between central and peripheral regulation of glucose metabolism in this experimental model. Since liver responses to caloric restriction were reversed upon refeeding, it was hypothesized that both liver and hypothalamus would be affected by the prevailing feeding regimen. Also, as our earlier work imposed caloric restriction and refeeding to adult rats from 12-pups litters, it was tested whether the findings of our previous research were triggered, or at least influenced, by litter size.

## Materials and Methods

### Experimental Groups

The procedures were approved by the Ethics Commission in the Use of Animals (CEUA 8401200317). All the animals were kept under controlled conditions of illumination (12 h light/12 h dark, lights on at 6 a.m.), temperature (22 ± 2°C) and air flow.

Pregnant Wistar rats were supplied by the central animal house of the Institution. Dams had free access to water and commercial rodent chow (Nuvital^®^; Nuvilab, Curitiba, Brazil) throughout gestation and lactation. One day after parturition, pups were culled in litters of 6 (group G6) or 12 pups (group G12). Males were preferably used; females were included when necessary to complete litter size. The remaining pups were given a lethal dose of anesthetic (thionembutal 120 mg/kg after lidocaine 5 mg/kg, i.p.). The same euthanasia was used for the dams and female pups upon weaning (21 days after birth), while male pups were assigned to the following conditions: G6L and G12L (free access to chow from weaning to 90 days of age); G6R and G12R (chow reduced by 50% from weaning to 90 days of age); G6RL and G12RL (chow reduced by 50% from weaning to 60 days of age and free access to chow until 90 days of age). The 50% reduction in the amount of chow (groups GR and GRL) was calculated based on the intake of GL of corresponding age and from the same litter size.

The rats were housed in groups of 3 in plastic boxes with water *ad libitum* until 90 days of age, when the experimental protocols were carried out. Body weight was recorded at 21 and 90 days of age. Naso-anal length was recorded at 90 days of age to calculate the body mass index (BMI, g/cm^2^). Each rat was used in a single protocol.

### Pyruvate Tolerance Test (PTT)

PTT was conducted to assess *in vivo* gluconeogenic capacity. Six rats of each group, after overnight fasting (14 h), were anesthetized (thionembutal 40 mg/kg after lidocaine 5 mg/kg, i.p.) and were given an i.p. injection of pyruvate (2 g/kg) dissolved in NaCl 0.9% (0.25 g/mL, injected volume of 1.25 mL/100 g) (Rodgers and Puigserver, 2007). Blood samples were obtained from a puncture on the tail at times 0, 5, 10, 15, 30, 45, and 60 min, with 0 min the time immediately prior to pyruvate injection. Blood glucose (mg/dL) was determined with test-strips and glucometer (Optium Exceed^®^; Abbott, São Paulo, Brazil). The percentage increase of blood glucose and the area under the curve (AUC) of blood glucose variation was calculated for each rat taking glucose at time 0 as baseline.

### Quantitative PCR of Hypothalamic Neuropeptides

The hypothalamus was removed from overnight-fasted rats (*n* = 6/group) after euthanasia (thionembutal 120 mg/kg after lidocaine 5 mg/kg, i.p.). Extraction of total RNA was made according to the method of TRIzol (Invitrogen, Carlsbad-CA, United States). For the production of cDNA the High Capacity cDNA Reverse Transcription kit (Applied Biosystems, Foster City-CA, United States) was employed, the final concentration of cDNA being 3.0 μg. This was diluted according to the necessary concentration for the efficient amplification of each gene. The real-time PCR reactions were carried out in the ABI Prism 7500 Sequence Detection System (Applied Biosystems) using TaqMan (Applied Biosystems). The GAPDH gene (TaqMan TM – Applied Biosystems) was used as endogenous control of the reaction, normalizing the expression of the genes of interest on the samples. Before the experiments of relative quantification of expression of any gene, the gene/target system was validated with the endogenous control GAPDH. Only efficiencies close to 100% were considered (Araujo et al., 2018). Individual values of gene expression for each group were divided by the average expression for G6L. Results were expressed as fold change.

### *In situ* Liver Perfusion

Liver glucose metabolism was assessed through the technique of *in situ* liver perfusion. Gluconeogenesis was investigated with alanine infusion, while residual glycogenolysis was stimulated by adrenaline. The rats (*n* = 8/group), after overnight fasting, were anesthetized (thionembutal 40 mg/kg after lidocaine 5 mg/kg, i.p.) and the portal vein and inferior cava vein were cannulated. The liver was perfused with Krebs-Henseleit buffer (KH, pH 7.4) in a non-recirculating system. The perfusion fluid was pumped through a membrane oxygenator saturated with carbogenic mixture (O_2_/CO_2_ 95/5%) and warmed at 37°C before entering the liver through the portal vein. Immediately after the beginning of the perfusion, the diaphragm was sectioned for euthanasia (Babata et al., 2014).

After 20 min of stabilization, samples of the effluent fluid were collected from the inferior cava vein every 5 min. During collection, the liver was sequentially perfused with KH (basal perfusion, 10 min), KH + the glycogenolytic agent adrenaline (1 μM, Adr1 period, 30 min), KH + the gluconeogenic substrate alanine (5 mM, Ala period, 30 min) and again KH + adrenaline (1 μM, Adr2 period, 20 min). These concentrations were established in previous experiments (Babata et al., 2014; Bataglini et al., 2017).

When the perfusion was finished, the liver and fat pads (retroperitoneal, periepididymal, mesenteric, and inguinal) were removed and weighed.

Samples of the effluent fluid were used to determine glucose and total nitrogen through enzymatic-colorimetric methods (commercial kits; GoldAnalisa, Belo Horizonte, Brazil) and lactate and pyruvate through enzymatic methods (Czok and Lamprecht, 1974; Gutmann and Wahlefeld, 1974), all expressed as μmol/min per g liver. The effluent amount of each compound in each period of the perfusion was expressed as AUC (μmol/g liver) after subtracting the output of the compound at the end of the previous period.

### Statistical Analysis

Resource equation value was 30–42, above the upper recommended value of 20. The surplus in each experiment was a precaution against unexpected setbacks. In addition, coefficient of variation was kept below 30% whenever possible. All the data sets are exhibited as mean ± SD and were subjected to Shapiro-Wilk and Kolmogorov-Smirnov normality tests. Comparisons were made through two-way ANOVA with Bonferroni *post hoc* test, except when stated otherwise. Statistical significance was set at 5% (*p* < 0.05). Statistical analyses and graphs were made in Prism^®^ 5.0 (GraphPad, San Diego-CA, United States).

## Results

### Biometric Records

At weaning, body weight of G12 (39.25 ± 4.48 g, *n* = 35) was 1.5x lower than that of G6 (57.67 ± 3.26 g, *n* = 32) (*p* < 0.05, *t*-test). Feeding regimen had a marked effect on the biometric data of the adult rats (Table 1), while litter size had significant effect only on body weight. No interaction was seen between feeding regimen and litter size. After caloric restriction until 90 days of age (G6R and G12R), body weight was 48–50% lower compared with their freely-fed peers (G6L and G12L, respectively). Rats refed after caloric restriction (GRL) had body weight 65–70% higher than the corresponding GR, although still lower (13%) than the corresponding GL. The significant effect of litter size on body weight (Table 1) was probably caused by the smaller values in the G12 groups compared with those of the G6 (Bonferroni *p* > 0.05).

**TABLE 1:** Biometric data of 90 d-old rats of groups G6L, G6R, G6RL, G12L, G12R, and G12RL.

	**G6L**	**G6R**	**G6RL**	**G12L**	**G12R**	**G12RL**	**Interaction**	**Litter size**	**Feeding regimen**
							**p (F), *Df* = 2**	**p (F), *Df* = 1**	**p (F), *Df* = 2**
BW (g)	366.6 ± 18.69	188.1 ± 12.73	318.6 ± 24.74	346.9 ± 21.69	181.3 ± 7.89	297.5 ± 25.07	0.548 (0.61)	**0.009** (7.63)	**<0.001** (310.2)
BMI (g/cm^2^)	0.56 ± 0.02	0.46 ± 0.02	0.58 ± 0.03	0.55 ± 0.01	0.46 ± 0.04	0.54 ± 0.04	0.259 (1.41)	0.156 (2.11)	**<0.001** (40.66)
VF (g%)	2.60 ± 0.62	1.31 ± 0.23	2.46 ± 0.88	2.20 ± 0.32	1.25 ± 0.25	2.25 ± 0.86	0.772 (0.26)	0.244 (1.40)	**<0.001** (13.78)
IF (g%)	0.74 ± 0.17	0.69 ± 0.12	0.84 ± 0.20	0.78 ± 0.12	0.67 ± 0.08	0.77 ± 0.07	0.513 (0.68)	0.641 (0.22)	0.052 (3.18)

**BW, body weight; BMI, body mass index; VF, visceral fat; IF, inguinal fat. Data are means ± SD; n = 6–8/group. Significant *p* values are shown in bold type.**

BMI (Table 1) calculated at 90 days of age also showed differences across the groups, but of smaller percentages than body weight because maximal naso-anal length range was 3 cm. Animals from GR had BMI 18% lower than the GL, and refeeding (GRL) reversed this value to that of GL.

Table 1 also shows the relative weight of the fat pads. Visceral fat pads (retroperitoneal+periepididymal+mesenteric) weighed 45% less in GR than in GL and 45–50% more in GRL compared with GR. On the other hand, the subcutaneous (inguinal) fat pad varied little across the groups, so that no difference due to feeding regimen or litter size attained significance.

### *In vivo* Blood Glucose Kinetics

The results of PTT are shown in Table 2. Feeding regimen had a marked effect on both variables. Individual percentage increases of blood glucose after pyruvate injection spanned a wide range within each group but were null or small in G6R and G12R compared with the corresponding GL. Percentages were high after refeeding (GRL), with an effect of litter size between G6L and G12L (Bonferroni, *p* < 0.01). Rats under caloric restriction (GR) showed ranges of blood glucose variation (indicated by the AUC in Table 2) smaller than their freely-fed (GL) or refed (GRL) peers. The interaction of litter size and feeding regimen was significant for groups G6L/G12L (Bonferroni, *p* < 0.05) and G6RL/G12RL (Bonferroni, *p* < 0.0001).

**TABLE 2:** Data of the pyruvate tolerance test of 90 d-old rats from groups G6L, G6R, G6RL, G12L, G12R, and G12RL.

	**G6L**	**G6R**	**G6RL**	**G12L**	**G12R**	**G12RL**	**Interaction**	**Litter size**	**Feeding regimen**
							**p (F), *Df* = 2**	**p (F), *Df* = 1**	**p (F), *Df* = 2**
AUC	1343 ± 181.9	81.25 ± 51.27	1697 ± 548.9	1886 ± 351.3	86.67 ± 66.63	853.4 ± 381.8	**<0.0001** (14.41)	0.3622 (0.86)	**<0.0001** (76.18)
	1133–1448	0–117.5	1095–2170	1363–2113	0–375	500–1243			
%	35.75 ± 11.64	0	18.12 ± 6.69	54.73 ± 16.66	7.53 ± 5.35	21.99 ± 6.95	0.1411 (2.01)	**0.0031** (10.36)	**<0.0001** (58.79)

**AUC, area under curve; %, percentage increase relative to basal blood glucose. Data are means ± SD and range (for AUC, bottom line); n = 5–6/group. Significant *p* values are shown in bold type.**

### Liver Glucose Metabolism

**FIGURE 1:**
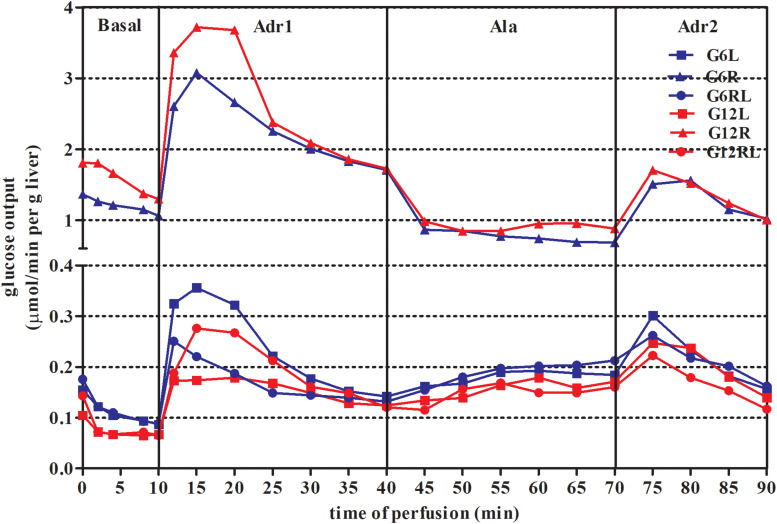
Glucose output during *in situ* liver perfusion of 90 d-old rats of groups G6L, G6R, G6RL, G12L, G12R, and G12RL. Data are means; *n* = 6–8/group.

*In situ* liver perfusion was carried out after 14 h of overnight fasting. Glucose output of the groups is shown in Figure 1, where the large total glucose output of G6R and G12R is striking.

**FIGURE 2:**
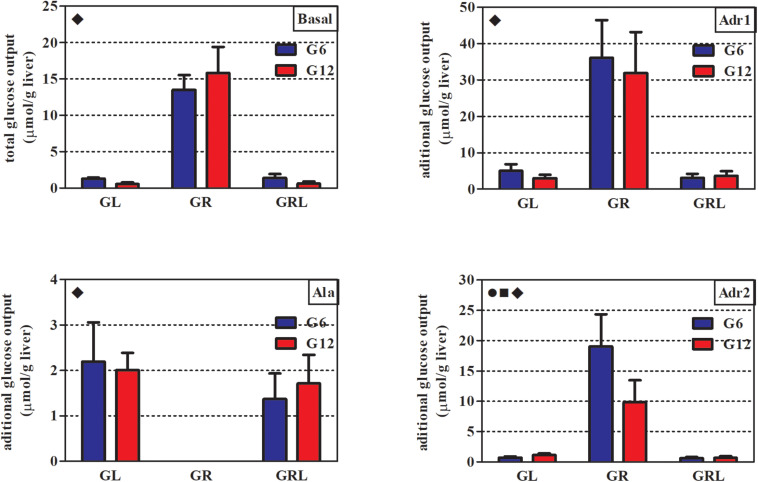
AUCs of glucose output during *in situ* liver perfusion of 90 d-old rats of groups G6L, G6R, G6RL, G12L, G12R, and G12RL. Adr1, first perfusion with adrenaline; Ala, perfusion with alanine; Adr2, second perfusion with adrenaline. Data are means ± SD; *n* = 6–8/group. 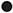, interaction effect; ■, litter size effect; ◆, feeding regimen effect; two-way ANOVA, *p* < 0.05.

In all the perfusion periods (basal, Adr1, Ala, and Adr2), feeding regimen had a markedly significant effect on glucose output (Table 3). As a consequence, the AUCs of glucose output of GL and GRL were similar, while both GR were quite different from the others (Figure 2).

**TABLE 3:** Summary of two-way ANOVA for the variables of Figures 2, 3.

**Variable**	**Interaction p (F), *Df* = 2**	**Litter size p (F), *Df* = 1**	**Feeding regimen p (F), *Df* = 2**
**Figure 2**			
Basal	0.0513 (3.29)	0.6475 (0.21)	**<0.0001** (256.3)
Adr1	0.6645 (0.41)	0.3698 (0.83)	**<0.0001** (91.04)
Ala	0.4680 (0.78)	0.7597 (0.09)	**<0.0001** (52.82)
Adr2	**<0.0001** (13.03)	**0.0024** (11.00)	**<0.0001** (108.0)

**Figure 3**			

NPY	0.3233 (1.18)	**0.0031** (10.67)	**0.0274** (4.15)
AgRP	**0.0206** (4.62)	**<0.0001** (56.10)	**<0.0001** (18.25)
POMC	**<0.0001** (19.66)	**<0.0001** (29.05)	0.1404 (2.16)
CART	**<0.0001** (23.72)	**0.0025** (11.45)	**0.0014** (8.73)

*Significant *p* values are shown in bold type.*

During the basal period, glucose output was about 10x higher in G6R and 28x higher in G12R compared with the GL of the same litter size. Refeeding resulted in values similar to those of GL. During the first adrenaline infusion (Adr1), G6R and G12R had additional glucose output – that is, above that of the basal period – about 7x (G6R) and 10.5x (G12R) higher than their respective GL and GRL. The animals under caloric restriction (GR) did not have additional glucose output during alanine infusion (Ala), in contrast to GL and GRL. Finally, during the second infusion of adrenaline (Adr2), additional glucose output of G6R was 26.5x higher than G6L, while that of the G12R was 8.7x higher than G12L. Additional glucose output during Adr2 was small in GL and GRL. For Adr2, both litter size and the interaction, in addition to feeding regimen, had a marked effect on the additional glucose output of the GR (Bonferroni *p* < 0.001 for G6R/G12R), as this was twice as high in G6R than in G12R.

The outputs of lactate, pyruvate and total nitrogen were determined only during alanine infusion (Ala, Figure 1) and are shown in Table 4. Pyruvate output did not differ across the groups. Lactate output was 6x lower in G6R than in G6L and 2x lower in G12R than in G12L. Feeding regimen, litter size and the interaction affected lactate output (Bonferroni *p* < 0.001 for G6RL/G12RL, which were different in lactate output by a factor of 2.7x). Nitrogen output of the refed groups was about 30% lower than in GL and GR, which probably caused the significant effect of feeding regimen shown in Table 4.

**TABLE 4:** AUCs of lactate, pyruvate, and nitrogen outputs during *in situ* liver perfusion of 90 d-old rats of groups G6L, G6R, G6RL, G12L, G12R, and G12RL.

**Output**	**G6L**	**G6R**	**G6RL**	**G12L**	**G12R**	**G12RL**	**Interaction**	**Litter size**	**Feeding regimen**
**μ mol/g liver**							**p (F), *Df* = 2**	**p (F), *Df* = 1**	**p (F), *Df* = 2**
Lactate	1.88 ± 0.65	0.30 ± 0.22	1.67 ± 0.51	1.40 ± 0.29	0.68 ± 0.13	0.62 ± 0.34	**0.0005** (9.84)	**0.0070** (8.37)	**<0.0001** (25.28)
Pyruvate	0.52 ± 0.71	0.41 ± 0.19	0.33 ± 0.12	0.54 ± 0.11	0.44 ± 0.12	0.52 ± 0.47	0.8164 (0.20)	0.5165 (0.43)	0.7216 (0.33)
Nitrogen	26.72 ± 4.73	23.66 ± 3.20	19.31 ± 5.12	22.65 ± 6.59	23.36 ± 4.77	16.15 ± 3.66	0.6095 (0.50)	0.1274 (2.46)	**0.0028** (7.21)

**Data are means ± SD; n = 6–8/group. Significant *p* values are shown in bold type.**

### Hypothalamic Neuropeptide Gene Expression

**FIGURE 3:**
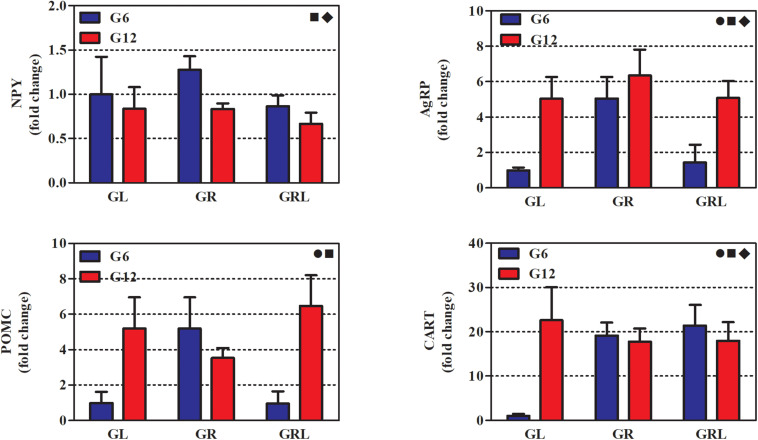
Gene expression of hypothalamic neuropeptides of 90 d-old rats of groups G6L, G6R, G6RL, G12L, G12R, and G12RL. NPY, neuropeptide Y; AgRP, agouti gene-related peptide; POMC, pro-opiomelanocortin; CART, cocaine- and amphetamine-related transcripts. Data are means ± SD; *n* = 4–6/group. 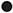, interaction effect; ■, litter size effect; ◆, feeding regimen effect; two-way ANOVA, *p* < 0.05.

Figure 3 shows the gene expression of neuropeptides from hypothalamic samples collected after 14 h of fasting. The results of the two-way ANOVA are in Table 3.

All neuropeptides were markedly affected by litter size; G6R had higher NPY than G12R (Bonferroni, *p* < 0.05). The levels of AgRP, POMC, and CART were higher in G12L than G6L, and AgRP and POMC were higher in G12RL than in G6RL (Bonferroni, *p* < 0.001 for both comparisons).

Except for POMC, all the other neuropeptides were also affected by feeding regimen. The interaction of the two factors was not significant only for NPY (Table 3). Fold changes were high in G6R for AgRP (5x), POMC (5x), and CART (19x); in G6RL, they were smaller for AgRP (1.4x) and POMC (1x), but not CART (21.4x). Fold changes for G12 were high, however, their range was narrower than G6 across feeding regimens (Figure 3): 0.6–0.8 for NPY, 5.0–6.3 for AgRP, 3.5–6.5 for POMC, 17.9–22.6 for CART.

## Discussion

One way of altering the post-natal nutrition in rodents is by changing litter size (i.e., the number of pups per litter). Consequently, rats from large litters had lower body weight at weaning than those from small litters. In large litters, the dam spends less time taking care of the pups. In addition, milk availability is higher in small litters, and the milk is richer in fat and calories than that of dams bearing large litters (Fiorotto et al., 1991; Bouret, 2012).

The timing (gestation/lactation), type (calorie/protein), and severity of restriction (dependent, e.g., of litter size) determine the outcome of perinatal sub-nutrition in the adult animal regarding growth, metabolism and physiology (Koletzko et al., 2005; Bouret, 2012; Picó et al., 2012). In this study, there was little difference in body weight or BMI of 90 d-old rats from the two litters (6 or 12 pups) kept under the same feeding regimen after weaning. In the same way, litters of both sizes under the same feeding regimen did not show marked differences in fat weight. Low body weight, BMI and fat weight were observed only in rats under 50% caloric restriction until 90 days of age (G6R and G12R alike) compared to those fed freely after weaning (G6L and G12L) or refed for 30 days (G6RL and G12RL). Clearly, these biometric parameters did not point to any later consequence of the initial (i.e., lactational) nutrition but instead reflected the nutritional condition of the adult animal.

The low weight of the visceral fats in the caloric-restricted groups is not surprising, since fat deposition is expected only when energy intake is higher than energy expenditure. However, refeeding (G6RL and G12RL) increased visceral fat only up to the point of equaling that of the GL. From this observation it can be suggested that the prolonged caloric restriction (from weaning to 60 days of age) did not trigger an accelerated compensatory accumulation of fat when chow was freely available in adult life (from 60 to 90 days of age), at least not for 30 days (Malta and Pedrosa-Furlan, 2010).

Visceral adipose tissue is more sensitive to catecholamine-stimulated lipolysis, especially during stress or fasting (Ravussin and Smith, 2002), and the caloric-restricted rats were constantly under these circumstances. In contrast, the inguinal adipose tissue did not undergo significant changes due to feeding regimens. As this tissue seems to be less harmful or even protective in what concerns metabolic impairments (Mauer et al., 2001), this observation may be relevant. It is argued that the inguinal adipose tissue would be a preferred site for lipid storage, protecting the organism against ectopic fat depots, e.g., liver and skeletal muscle, where fat would have greater harmful potential (Weiss et al., 2007).

Since 2007, our research group has been studying the effects of caloric restriction on liver glucose metabolism in rats from different litter sizes. For the first time, in this investigation a sequential liver perfusion was used: basal, with glycogenolytic agent (Adr1), with gluconeogenic substrate (Ala) and again with glycogenolytic agent (Adr2). Given that the liver remains viable for up to 3 h as long as the infusion of properly oxygenated and warmed buffer is constant, the reduction in the number of experimental animals is substantial, and the results from different compounds are obtained from the same liver, creating an individualized profile of the organ.

For the most part, results here match those of previous studies (Malta et al., 2010; Vitoriano et al., 2011; Babata et al., 2014; Garcia et al., 2017): rats under caloric restriction, even after overnight fasting, showed high basal glucose output, responded markedly to the glycogenolytic action of adrenaline and did not increase glucose output in the presence of the gluconeogenic substrate alanine. This metabolic profile is similar to that found in rats in prandial or early post-prandial state – that is, subjected to perfusion soon after feeding (Babata et al., 2014).

Total glucose output was much higher in GR than in GL and additional glucose was released during Adr1 and Adr2. This can be an indirect indication of the presence of glycogen in the hepatocytes. Although glycogen content did not differ between GL and GR in a previous study (Vitoriano et al., 2011), basal and adrenaline-stimulated glucose output are markedly reduced after an incremental exercise session (Babata et al., 2014), which promotes energy discharge of the liver and releases glucose to the bloodstream to sustain the intense energy demand of the working muscles (Trefts et al., 2015); indirectly, these observations suggest the persistence of glycogen in the GR hepatocytes despite overnight fasting.

AUCs of glucose output, except for the basal period, refer to additional output, i.e., output of the current perfusion period minus that of the previous one. Therefore, although total glucose output has been much higher in the caloric-restricted groups (G6R and G12R) during the whole perfusion, no additional increase took place during alanine infusion (Ala).

The insignificant additional glucose output during Ala in the GR corresponded to the negligible or much reduced hyperglycemic effect of pyruvate *in vivo* during the PTT. This suggests that the gluconeogenic substrate (alanine in liver perfusion) or its immediate product (pyruvate in PTT) must have followed a pathway other than gluconeogenesis. Alternatively, glucose produced from these compounds was not released by the liver to the bloodstream (during PTT) or effluent fluid (during perfusion). Both pyruvate and alanine are at the crossroad of several metabolic routes, and the predominant destination of each depends upon the metabolic status of the tissue and/or organism at that time (Adeva et al., 2013). Oxidation of alanine carbon skeleton (i.e., pyruvate) for energy release (because of a decreased cytosolic NADH/NAD^+^ ratio) or indirect glycogen synthesis (from lactate, mostly at the periportal zone of the liver acini) (Ghafoory et al., 2013) are plausible destinations. These would explain the large total glucose output, unaltered pyruvate and nitrogen outputs (the latter resulting from deamination and ureagenesis) and decreased lactate output (the product of pyruvate reduction) in the caloric-restricted groups.

The reduced output of nitrogen in the GRL is logical, considering that these animals were still recovering body weight after a long period (40 days) of caloric restriction. Even during circadian fasting, liver nitrogen output, either as ammonia or urea, is decreased, nitrogen being exported by the liver preferably as glutamine (Lopez et al., 1998).

The hypothalamus has a significant role in several aspects of energy metabolism, such as food intake, energy expenditure, peripheral insulin sensitivity, and glucose homeostasis (Bouret, 2012, 2017; Picó et al., 2012; Spencer, 2013; De Solis et al., 2016; Ruud et al., 2017; Palou et al., 2018). Therefore, it would be expected that there was a parallel between liver glucose metabolism and hypothalamic neuropeptidergic expression. Surprisingly, this did not happen in either litter size. The analyzed neuropeptides did not show a pattern of expression that matched – and hence could explain – the metabolic changes of the liver. In other words, none of the hypothalamic neuropeptides was altered in either GR group in a manner that corresponded to the liver modifications.

Nevertheless, a few observations concerning the gene expression of these neuropeptides deserve some comments. First, there was an increase in the expression of NPY, AgRP, POMC, and CART in the G6R compared with G6L. Second, a low expression after refeeding (G6RL) was recorded for some neuropeptides (NPY, AgRP, and POMC, which returned to G6L levels), but not for CART (which remained as high as G6R). And finally, gene expression of these neuropeptides in rats from large litters (G12) was less affected by feeding regimen, but was higher than in G6 (except for NPY). These observations indicate that hypothalamic responsiveness is complex and depends upon litter size, feeding regimen and/or both. In contrast, liver responsiveness was clearly dependent on feeding regimen; litter size had only punctual influence.

Change of hypothalamic gene expression and functioning due to lactational manipulations are often reported (Bouret, 2012, 2017; Picó et al., 2012; Spencer, 2013; De Solis et al., 2016) and correlated with permanent (adult) changes of energy metabolism, adiposity and food intake. The hypothalamic organization takes place both in the intra-uterine environment and after birth, starting at mid-gestation and lasting for at least 2 weeks after birth in rats (Remmers and Delemarre-Van De Wall, 2011; Bouret, 2012, 2017). Intra-hypothalamic synaptic contacts (and possibly those of the hypothalamus with other brain regions) mature during even longer periods that extend through adult life (Bouret, 2012). Hypothalamic plasticity induced by nutritional factors at the beginning of development seems to involve adjustments at several levels: size of hypothalamic areas; types, numbers and inter-connections among neurons; and neuronal response to indicators (such as leptin, insulin, ghrelin and glucocorticoids) of acute and chronic energy status (Spencer, 2013; De Solis et al., 2016; Ruud et al., 2017; Dodd et al., 2018). In this context, it is reported that leptin has a fundamental role in the maturation and activation of hypothalamic neurons, especially in the ARC, during development. In rodents, there is a surge of this hormone in the first post-natal days, this being a key developmental signal that shapes the architecture of hypothalamic circuits controlling energy balance (Bouyer and Simerly, 2013; Bouret, 2017). Both maternal caloric restriction and large litters during lactation decrease the circulating levels of leptin in the pups (Bouret, 2012, 2017; Picó et al., 2012; De Solis et al., 2016; Palou et al., 2018). Although other factors might be in action, this alteration in the lactational surge of leptin could be related to the different expression (and possibly, effect) of the neuropeptides between the two litter sizes of this study.

NPY/AgRP and POMC/CART neurons are populations with dual actions on food intake and other aspects of energy metabolism (Ruud et al., 2017). The simultaneous high level of these neuropeptides in G6R is difficult to interpret based solely on the expression of these substances. Despite reflecting structural changes of the hypothalamus (Picó et al., 2012), quantification of gene expression, *per se*, does not take into account that other functional aspects – e.g., which neurons are active, their individual responsiveness to peripheral factors related to energy homeostasis and their connections – are relevant for the hypothalamic effects on energy metabolism of peripheral tissues. For instance, it is reported that different subsets of POMC- and AgRP-expressing neurons exhibit distinct transcriptional responses to variable energy states (Dodd et al., 2018). Therefore, an increased or decreased gene expression is only part of the scenario of the hypothalamic functioning in a given situation.

It is unlikely that the sustained high level of CART has been “programmed” by the initial nutrition of G6RL. Instead, our supposition is that, given a longer period or refeeding, CART expression would be restored to the level of G6L, as AgRP and POMC did after 30 days. In this way, a dynamic component of nutrition-dependent hypothalamic neuropeptidergic expression would be in motion in the adult rat, as much as the hypothalamus responds dynamically to the perinatal nutritional conditions (Bouret, 2012, 2017; Picó et al., 2012).

Though the neuropeptidergic scenario is insufficient to outline a more detailed picture of hypothalamic functioning in the experimental model of this study, it seems reasonable to state that, in small litters, neuropeptide expression was responsive to the actual feeding regimen, instead of showing a constant pattern linked to earlier (lactational) nutritional experiences. In favor of this possibility are the differing levels of neuropeptides in G6L, G6R, and G6RL. On the other hand, neuropeptide expression in large litters (G12) seems to indicate an hypothalamic activity established at the initial stages of post-natal development and less responsive to post-lactational nutrition.

In the previous studies of the research group, the small (6 pups) litters were included for biometric comparison with the large ones (12 pups), but only the latter were subjected to caloric restriction and/or refeeding (Malta et al., 2010; Vitoriano et al., 2011; Babata et al., 2014; Garcia et al., 2017). Therefore, the liver metabolic profile could be the result of some residual influence of the limited food supply during lactation and, as such, might not be present if animals from small litters were food restricted. In the present study, both litters (G6 and G12) were subjected to the same post-lactational feeding regimens to explore this possibility. The *in situ* liver perfusion showed that liver glucose metabolism observed in caloric restriction (1) was hardly influenced by litter size and (2) was reversed to that of freely-fed rats by 30 days of refeeding.

In conclusion, both liver glucose metabolism and hypothalamic neuropeptide gene expression are influenced by nutritional condition. However, while lactational nutrition (determined by litter size) did not affect liver metabolism of adult animals, the hypothalamus revealed such effect, given that the feeding manipulations modulated neuropeptide gene expression differently in rats from the two litter sizes. Thus, liver glucose metabolism of rats under caloric restriction was not primarily linked to hypothalamic circuiting, but to the prevailing nutritional condition.

## Data Availability Statement

The raw data supporting the conclusions of this article will be made available by the authors, without undue reservation, to any qualified researcher.

## Ethics Statement

The animal study was reviewed and approved by Ethics Comission on Animal Experimentation (CEUA), State University of Maringá, Brazil.

## Author Contributions

IM, LY, RG, and MP conceived and planned the experimental design, analyzed the data, and revised the final version of the manuscript. Experiments were conducted by IM, LY, RG, RS, VR, CB, and SA. Manuscript was written by IM, LY, and MP.

## Conflict of Interest

The authors declare that the research was conducted in the absence of any commercial or financial relationships that could be construed as a potential conflict of interest.
